# Recurrence of Pterygium after Pterygium Excision with Stem Cell Graft and Amniotic Membrane Graft: A Comparison

**DOI:** 10.7759/cureus.6535

**Published:** 2020-01-01

**Authors:** Syed Ahmer Hussain, Kamran Haider Shaheen, Muhammad Saad Ullah, Aamir Furqan

**Affiliations:** 1 Ophthalmology, District Headquarter Teaching Hospital, Dera Ghazi Khan, PAK; 2 Anesthesiology, Chaudhry Pervaiz Elahi Institute of Cardiology, Multan, PAK

**Keywords:** pterygium, excision, stem cell graft, amniotic membrane graft, recurrence, edema, hemorrhage

## Abstract

Study objective

The objective of this study was to compare pterygium excision with amniotic membrane graft and that with stem cell graft in terms of pterygium recurrence, using a quasi-experimental study design. This study was conducted at the department of ophthalmology at Nishtar Hospital, Multan, Pakistan from January to September 2019.

Methodology and results

A total of 214 patients who presented to the outpatient section at the department of ophthalmology were included in this study. A lottery method was used to divide the patients into two equal groups: A and B. Patients in group A underwent pterygium excision with amniotic membrane graft, and patients in group B underwent pterygium excision with stem cell grafts. Follow-ups were planned for the third day, the second week, the first month, the third month, and the sixth month postoperatively. Frequency and percentage were calculated for qualitative variables and for quantitative data. Mean and standard deviation were calculated, and a p-value of <0.05 was considered significant. Postoperative complications (i.e., graft edema, hemorrhage, and recurrence) in group A were observed as n = 0 (0%), n = 11 (10.3%), and n = 15 (14%), respectively. Postoperative complications (i.e., graft edema, hemorrhage, and recurrence) in group B were observed as 15%, 3.7%, and 12.1%, respectively. Statistically, the difference for graft edema was significant (p = 0.000).

Conclusion

Stem cell grafting after pterygium excision was not associated with any major complications postoperatively. Stem cell grafting is better in terms of cosmetic appearance and has less recurrence rate as compared to amniotic membrane transplantation.

## Introduction

Overgrowth of fibrovascular tissue of bulbar conjunctiva onto the cornea across the limbus is known as pterygium. As far as the epidemiology of pterygium is concerned, its incidence varies with various geographical regions and is found more commonly in people of certain populations. The causative factors of pterygium include excessive light exposure, heat, dryness, dust, and wind. Research shows that it is twice as common among people working outdoors and five times less common among people who wear sunglasses in outdoor working environments [1]. According to one study, the incidence of pterygium increased 1.5 times in people working in outdoor settings, while the risk was decreased substantially in darker populations and in people who had the habit of using sunglasses [2]. Among the factors recognized in the pathogenesis of pterygium, ultraviolet radiation is most common, but the main mechanism through which it results in pterygium formation is unclear [3,4].

Another factor that was found to be affecting the pathogenesis of the pterygium formation is a heparin-binding epidermal growth factor, which has the potential to stimulate altered cellular anchorage independence and growth [5].

The main aim of the surgical removal of the pterygium is to prevent a recurrence, complete excision, and rehabilitation of the integrity of the ocular surface. Numerous techniques used independently or combined with each other are being applied in the treatment of pterygium. The most common postoperative complication of all these procedures is the recurrence of the pterygium. Some reports suggest that the recurrence rate is almost 30-70% [6]. Some of the techniques used for excision of pterygium include excision with an injection of mitomycin C (antimetabolite adjuvants), bare sclera excision, amniotic membrane transplant after excision, excision along with simple closure of the conjunctiva, and excision with the use of conjunctival autograft [7,8]. A decrease in recurrence by almost 5-12% has been seen with the use of certain modalities such as the use of antimetabolite agents and radiation therapy. Even though these treatments decreased the incidence of recurrence of pterygium, they were associated with a number of other complications such as scleral necrosis, uveitis, cataracts, secondary glaucoma, secondary endophthalmitis, and corneal perforation [9].

Conjunctival autograft was first used in 1985 for the management of advanced or recurrent pterygium [10]. Even though this surgical technique is time-consuming, it has a lower rate of recurrence with similar efficacy as other surgical techniques. It is also devoid of any dangerous side effects. The chance of recurrence of pterygium after removal is 50% in the first four months and 97% within the first year, as shown by the survival curve analysis. The role of limbal stem cells has also been reported in the pathogenesis of pterygium in recent studies. Researchers have suggested that a healthy limbus protects against conjunctival overgrowth [10].

Anatomically, pterygium is three-sided and is often found on the nasal side rather than on the temporal side. The symptoms of pterygium include vision loss, ocular irritation, and hyperemia. There are multiple studies in the literature where different modalities have been used to prevent the recurrence of pterygium, but the safety and efficacy of these techniques require further research. The main purpose of this study is a comparison between pterygium excision with the transplantation of amniotic membrane and that with stem cell autograft in terms of the recurrence of the condition. Since there is no substantial local research on this topic so far, we hope that our findings will help to raise awareness so that better treatment plans can be adopted to avoid the recurrence of pterygium.

## Materials and methods

We conducted a quasi-experimental study in the department of ophthalmology in Nishtar Hospital, Multan from January to September 2019. After receiving ethical approval from the hospital ethics board, informed consent was obtained from the patients. A study conducted by Qayyum et al. was used to calculate the sample size by using openepi.com [11]. A total of 214 patients who presented to the outpatient section were included in this study. Inclusion in the study was based on the following criteria: patients belonging to either gender, aged more than 18 years who presented with pterygium of grade 2 to 3 with reports of discomfort, disfigurement, and visual impairment. A nonprobability consecutive type of sampling technique was used to collect the sample size. Patients younger than 18 years who were unwilling to participate in the study, those with systemic disease, a pterygium of grade <2, those with foreign bodies in their eyes, or those with other eye diseases such as cataracts or glaucoma were excluded. Informed consent was obtained from all patients before including them in the study. A lottery method was used to divide the patients into two equal groups: groups A and B. Patients in group A underwent pterygium excision with amniotic membrane graft, and patients in group B underwent pterygium excision with stem cell grafts.

The amniotic graft was taken from the human placenta after human immunodeficiency virus (HIV) and hepatitis B and C screening, while the stem cell autograft was taken from the superior limbus. All procedures were performed by a consultant ophthalmologist with more than 10 years of experience. All data were collected by the researcher himself. Postoperatively, topical steroids and antibiotics were prescribed with a dosage of four times per day for one month with tapering, and an eye pad was applied for 72 hours. Follow-up was planned for the third day, the second week, the first month, the third month, and the sixth month postoperatively. Data were subjected to statistical analysis using IBM SPSS Statistics for Windows, Version 23.0 (IBM Corp, Armonk, NY). For quantitative data, mean and standard deviation were calculated, while frequency and percentage were calculated for qualitative variables such as levels of pterygium and gender. A p-value of <0.05 was considered significant.

## Results

A total of 214 patients (age range: 18-58 years) were included and underwent pterygium excision. The mean age of group A was 40 (±5) years. There were more male patients (n = 57, 53.3%) than female patients (n = 50, 46.7%). The mean age of group B was 41 (±5) years, and this group also had more male patients (n = 75, 70.1%) than female patients (n = 32, 29.9%). Statistically, the difference was significant for gender (p = 0.011; Table 1).

Pterygium grades 2 and 3 in group A were noted as n = 40 (37.4%) and n = 67 (62.6%), respectively, while pterygium grades 2 and 3 in group B were noted as n = 51 (47.7%) and n = 56 (52.3%), respectively. Graft edema, hemorrhage, and recurrence in group A were observed as n = 0 (0%), n = 11 (10.3%), and n = 15 (14%), respectively. Graft edema, hemorrhage, and recurrence in group B were observed as n = 16 (15%), n = 4 (3.7%), and n = 13 (12.1%), respectively. Statistically, the difference for graft edema was significant (p = 0.000; Table 2).

**Table 1:** Demographic characteristics of the patients n: number of patients *standard deviation

Variables	Group A, n = 107	Group B, n = 107	P-value
Age (years)	40 (±5)*	41 (±5)*	0.589
Gender			
Male	n = 57 (53.3%)	n = 75 (70.1%)	0.011
Female	n = 50 (46.7%)	n =32 (29.9%)	

**Table 2:** Outcomes by group n: number of patients

		Group A, n = 107	Group B, n = 107	P-value
Pterygium grade	Grade 2	n = 40 (37.4%)	n = 51 (47.7%)	0.128
	Grade 3	n = 67 (62.6%)	n = 56 (52.3%)	
Postoperative complications	Graft edema	n = 0 (0%)	n = 16 (15%)	0.000
	Hemorrhage	n = 11 (10.3%)	n = 4 (3.7%)	0.061
	Recurrence	n = 15 (14%)	n = 13 (12.1%)	0.685

## Discussion

Pterygium is a multifactorial degenerative disease [12]. Removal of pterygium via excision techniques is associated with postoperative complications and a high rate of recurrence, which can be difficult to control. Recurrence has an underlying mechanism that involves trauma, which leads to inflammation and propagation of fibroblasts and deposition of extracellular matrix [13]. According to our study, men more commonly presented with pterygium than women, which may be a result of the typical gender distribution of work in outdoor settings.

A study by Nakamura et al. showed no recurrence of pterygium after excision within the follow-up period, which contrasts with the results of our study [14]. In other studies, many other side effects apart from recurrence have been reported, such as cataract formation, scleral ulceration, and glaucoma development [15]. In the current study, apart from recurrence, minor postoperative complications have been observed, such as graft edema and hemorrhage. Studies have shown that conjunctiva and limbus play an integral role in the maintenance of corneal epithelium [16]. The results of our study are supported by multiple previous studies published in the literature [17,18].

Furthermore, conjunctival grafts have a better yield in terms of pterygium recurrence as well as overall recurrence time as conjunctival grafts, including limbus stem grafts, inhibit the effect on the remaining abnormal tissue and help in restoration of the limbal barrier with the help of limbal stem cells, resulting in reduced frequency and duration of pterygium recurrence [19,20]. A study conducted by Soliman et al. showed that, after stem cell grafting, recurrence of pterygium growth was only present in two cases (4.75%) [10].

## Conclusions

This study compared the rate of recurrence of pterygium with excision with transplantation of amniotic membrane against stem cell autograft. A total of 214 patients were included in this study, and they were assigned to two treatment groups via a lottery. After receiving either pterygium excision with amniotic membrane graft or pterygium excision with stem cell grafts, the patients were monitored via follow-ups several times up to six months postoperatively. It was found that stem cell grafting after pterygium excision was not associated with any major complications postoperatively. Stem cell grafting is better in terms of cosmetic appearance and has a lower recurrence rate as compared to amniotic membrane transplantation.
